# Towards End-to-End Acoustic Localization Using Deep Learning: From Audio Signals to Source Position Coordinates

**DOI:** 10.3390/s18103418

**Published:** 2018-10-12

**Authors:** Juan Manuel Vera-Diaz, Daniel Pizarro, Javier Macias-Guarasa

**Affiliations:** Department of Electronics, University of Alcalá, Campus Universitario s/n, Alcalá de Henares, 28805 Madrid, Spain; manuel.vera@edu.uah.es (J.M.V.-D.); daniel.pizarro@uah.es (D.P.)

**Keywords:** acoustic source localization, microphone arrays, deep learning, convolutional neural networks

## Abstract

This paper presents a novel approach for indoor acoustic source localization using microphone arrays, based on a Convolutional Neural Network (CNN). In the proposed solution, the CNN is designed to directly estimate the three-dimensional position of a single acoustic source using the raw audio signal as the input information and avoiding the use of hand-crafted audio features. Given the limited amount of available localization data, we propose, in this paper, a training strategy based on two steps. We first train our network using semi-synthetic data generated from close talk speech recordings. We simulate the time delays and distortion suffered in the signal that propagate from the source to the array of microphones. We then fine tune this network using a small amount of real data. Our experimental results, evaluated on a publicly available dataset recorded in a real room, show that this approach is able to produce networks that significantly improve existing localization methods based on *SRP-PHAT* strategies and also those presented in very recent proposals based on Convolutional Recurrent Neural Networks (CRNN). In addition, our experiments show that the performance of our CNN method does not show a relevant dependency on the speaker’s gender, nor on the size of the signal window being used.

## 1. Introduction

The development and scientific research of advanced perceptual systems has notably grown during the last decades, and has experienced a tremendous rise in recent years due to the availability of increasingly sophisticated sensors, the use of computing nodes with higher and higher computational power, and the advent of powerful algorithmic strategies based on deep learning (all of them actually entering the mass consumer market). The aim of perceptual systems is to automatically analyze complex and rich information taken from different sensors in order to obtain refined information on the sensed environment and the activities being carried out within them. The scientific works in these environments cover research areas ranging from basic sensor technologies to signal processing and pattern recognition. They also open the pathway to the idea of systems being able to analyze human activities, providing us with advanced interaction capabilities and services.

In this context, the localization of humans (being the most *interesting* element for perceptual systems) is a fundamental task that needs to be addressed so that the systems can actually start to provide higher level information on the activities being carried out. Without a precise localization, further advanced interactions between humans and their physical environment cannot be fulfilled successfully.

The scientific community has devoted a huge amount of effort to building robust and reliable indoor localization systems based on different sensors [1,2,3]. Non-invasive technologies are preferred in this context, so that no electronic or passive devices need to be carried by humans for localization. The two non-invasive technologies that have been mainly used in indoor localization are those based on video systems and acoustic sensors.

This paper focuses on audio-based localization from unknown wide-band audio sources (e.g., the human voice) captured by a set of microphone arrays placed in known positions. The main objective of the paper is to directly use the signals captured by the microphone arrays to automatically obtain the position of the acoustic source detected in the given environment.

Even though there have been a lot of proposals in this area, Acoustic Source Localization (ASL) is still a hot research topic. This paper proposes a Convolutional Neural Network (CNN) architecture that is trained end-to-end to solve the acoustic localization problem. Our CNN takes the raw signals captured by the microphones as input and delivers the 3D position of the acoustic source as its output.

The idea of using neural networks for sound processing is not new and has gained popularity in recent years (especially for speech recognition [4]). In the context of ASL, deep learning methods have been recently developed [5,6,7,8,9,10,11,12,13,14,15,16,17,18]. Most of these works focus on obtaining the Direction of Arrival (DOA) of the acoustic source. They also feed the network with feature vectors extracted from the audio signals. To the best of our knowledge, this is the first work in the literature that directly uses the speech signal as input and aims to directly estimating the source position coordinates in the room in three-dimensional space. The avoidance of hand-crafted features has been proven to increase the accuracy of classifications and regression methods based on Convolutional Neural Networks in other fields, such as in computer vision [19,20].

Our proposal is evaluated using both semi-synthetic and real data, and outperforms traditional solutions based on Steered Response Power (*SRP*) [21] (that are still being actively used in state-of-the-art systems [22,23,24,25]), and also shows better results than a very recent proposal based on a Convolutional Recurrent Neural Network [18].

The rest of the paper is organized as follows. Section 2 includes a review study of the state-of-the-art in acoustic source localization with special emphasis on the use of deep learning approaches. Section 3 describes the CNN-based proposal, with details on the training and fine tuning strategies. The experimental work is detailed in Section 4, and Section 5 summarizes the main conclusions and contributions of the paper and gives some ideas for future work.

## 2. State of the Art

The literature contains many approaches to address the acoustic source localization (ASL) problem. According to the classical literature review on this topic, these approaches can be broadly divided into three categories [26,27]: time delay based, beamforming based, and high-resolution spectral estimation based methods. This taxonomy relies on the fact that ASL has traditionally been considered a signal processing problem based on the definition of a signal propagation model [26,27,28,29,30,31,32,33,34], but, more recently, the range of proposals in the literature has also considered strategies based on exploiting the optimization techniques and mathematical properties of related measurements [35,36,37,38,39], and also the use of machine learning strategies [40,41,42], aimed at obtaining direct mapping from specific features to source locations [43], an area in which deep learning approaches are starting to be applied and that will be further described later in this section.

Time delay based methods (also referred to as *indirect methods*), compute the time difference of arrivals (TDOAs) across various combinations of pairs of spatially separated microphones, usually using the Generalized Correlation Function (GCC) [28]. In a second step, the TDOAs are combined with knowledge of the microphones’ positions to generate a position estimation [26,44].

Beamforming based techniques [23,27,30,34] attempt to estimate the position of the source by optimizing a spatial statistic associated with each position, such as in the Steered Response Power (*SRP*) approach, in which the statistic is based on the signal power received when the microphone array is steered in the direction of a specific location. *SRP-PHAT* is a widely used algorithm for speaker localization that is based on beamforming and was first proposed in Ref. [21] (although the formulation is virtually identical to the *Global Coherence Field* (GCF) described in  Ref. [45]), it combines the robustness of the SRP approach with Phase Transform (PHAT) filtering, which increases the robustness of the algorithm to signal and room conditions, making it an ideal strategy for realistic speaker localization systems [31,32,46,47,48]. There are other beamforming based methods such as the Minimum Variance Distortionless Response (MVDR) [33], which is the most widely used adaptive beamformer.

Regarding spectral estimation based methods, the multiple signal classification algorithm (MUSIC) [49] has been widely used, as it is able to handle arbitrary geometries and multiple simultaneous narrowband sources. MUSIC’s performance can be affected when the task has a low signal-to-noise ratio and by reverberant environments [27]. It also requires a good estimate of the number of active sources in the multiple source scenario.

In the past few years, deep learning approaches [50] have taken the lead in different signal processing and machine learning fields, such as computer vision [20,51] and speech recognition [52,53,54], and, in general, in any area in which complex relationships between observed signals and the underlying processes generating them need to be discovered.

The idea of using neural networks for ASL is not new. Back in the early nineties and in the first decade of the current century, works such as Refs. [40,55,56] proposed the use of neural network techniques in this area. However, an evaluation of realistic and extensive datasets was not viable at this time, and the proposals were somewhat limited in scope.

With the advent and huge increase of applications of deep neural networks in all areas of machine learning, promising works have also been proposed for ASL [5,6,7,8,9,10,11,12,13,14,15,16,17,18,57,58,59,60,61]. This is mainly due to the sophisticated capabilities and more careful implementation details of network architectures and the availability of advanced hardware architectures with increased computational capacity.

The main differences between the different proposals for the use of neural networks for ASL reside in the architectures, input features, network output (target), and the experimental setup (using real or simulated data).

With respect to the information given to the neural network, several works have used features that are physically related to the ASL problem. Some of the proposals have used features derived from the GCC or related functions which actually make sense, as these correlation function are closely related to the TDOAs which are used in traditional methods to generate position estimations. The published works have used either the GCC or GCC-PHAT coefficients directly [6,17], as well as features derived from them [11,58] or from the correlation matrix [5,60], or even combined with others, such as cepstral coefficients [9]. Other works have focused on exploiting binaural cues [13,57,59], and even trying to discover binaural features using a CNN [16]. Others have employed narrowband SRP values [14]. The latter approach goes one step further from correlation related values, as the SRP function actually integrates multiple GCC estimations in such a way that acoustic energy maps can be easily generated from them.

As opposed to the previously described works that used refined features that are directly related to the localization problem, others used frequency domain features directly [8,12,18,61]. In some cases, these were generated from spectrograms of general time–frequency representations [7,10]. These approaches represent a step forward compared with previous ones, as they give the network the responsibility of automatically learning the relationships between spectral cues and location-related information [15]. In this last reference, the authors combined both strategies, as they used spectral features but calculated them in a cross-spectral fashion, that is, they combined the values from all of the available microphones in the so-called Cross Spectral Map (CSM).

In none of the referenced works did the authors use the raw acoustic signal directly, and we are interested in evaluating the capabilities of CNN architectures in directly exploiting this raw input information. At this point, we must mention that the works using spectral features directly derived from linear and fully recoverable transforms [7,8,10,12,18,61], such as the STFT spectrum (thus containing the same amount of information than the time-domain signals), also used raw acoustic information. We do not claim that the use of time domain signals is better than using frequency domain, or cross-spectral features, but we want to exploit the windowed time domain signal without further preprocessing as an input to the network to assess whether its feasibility is a valid input feature in the ASL task.

Regarding the estimation target, most works have been oriented towards estimating the Direction of Arrival (DOA) of the acoustic sources [6,7,11,12,14,58], or the DOA-related measurements, such as the azimuth angle [13,57,59,61], the elevation angle [16], or the position bearing and range [9]. Some of the proposals posed the problem not as a direct estimation (regression) but as a classification problem among a predefined set of possible position related values [5,8,10,60,61] (azimuth, positions in a predefined grid, etc.). Other works tried to estimate a *clean* acoustic source map [15] or to learn time frequency masks as a preprocessing stage prior to ASL [62].

In only two of the referenced works [17,18] the authors try to directly find out the coordinate values of the acoustic sources. In Ref. [17], the source coordinates were estimatedin a bidimensional space, and in Ref. [18], a three-dimensional space was considered, but the estimated position was calculated as the *x*, *y*, and *z* axis coordinates of the DOA on a unit sphere around the microphone. In our proposal, we are again interested in further evaluating the capabilities of CNN architectures to directly generate the generic cartesian coordinates in a full three-dimensional space.

With respect to the number of active sources considered in the ASL task, most works have considered the single source case, but in the last couple of years, the multi-source localization scenario has also been addressed [7,12,18]. We focus on the single source case, as our target is to evaluate the feasibility of the end-to-end approach by estimating the three-dimensional cartesian coordinates of the source position.

Finally, regarding the experimental setup, most previous works used simulated data either for training or for training and testing [5,6,7,8,10,11,13,14,15,16,57,58,59,60,61,62], usually by convolving clean (anechoic) speech with impulse responses (room, head-related, or DOA-related (azimuth, elevation)). Only some of them actually used real recordings [9,11,14,17,57,58], which in our opinion is a must to be able to assess the actual impact of the proposals under real conditions.

So, in this paper, we describe a CNN architecture in which we directly exploit the raw acoustic signal to be provided to the neural network, with the objective of directly estimating the coordinates of the three-dimensional position of an acoustic source in a given environment. This is the reason why we refer to this strategy as end-to-end, considering the full coverage of the ASL problem. The proposal is evaluated using both semi-synthetic and real data from a publicly available database.

## 3. System Description

### 3.1. Problem Statement

Our system obtains the position of an acoustic source from the audio signals recorded by an array of *M* microphones. Given a reference coordinate origin, the source position is defined with the 3D coordinate vector $s={\left(s_{x}\;\;s_{y}\;\;s_{z}\right)}^{⊤}$. The position of the microphones are known, and they are defined with the coordinate vectors $m_{i}={\left(m_{i,x}\;\;m_{i,y}\;\;m_{i,z}\right)}^{⊤}$ with i=1,…,M. The audio signal captured from the *i*th microphone is denoted by $x_{i}\left(t\right)$. This signal is discretized with a sampling frequency $f_{s}$ and is defined as $x_{i}\left[n\right]$. For simplicity, we assume that $x_{i}\left[n\right]$ is of finite length with *N* samples. This corresponds to a small audio window with a duration of $w_{s}=N/f_{s}$, which is a design parameter in our system. We denote the vector containing all time samples of the signal as $x_{i}$:

(1) $$x_{i}={\left(\begin{array}{ccc} x_{i}\left[0\right] & \ldots & x_{i}\left[N-1\right] \end{array}\right)}^{⊤}.$$

The problem that we seek to solve is to find the following regression function (*f*):

(2) $$s=f\left(x_{1},\ldots ,x_{M},m_{1},\ldots ,m_{M}\right),$$

that obtains the speaker’s position given the signals recorded from the microphones.

In classical simplified approaches, *f* is found by assuming that signals received from different microphones mainly differ by a delay that depends on the relative position of the source with respect to the microphones. However, this assumption does not hold in environments where the signal is severely affected by the effects of reverberation due to multi-path propagation and the presence of diffuse and ambient noise.

Given the aforementioned effects and the random nature of the audio signal, the regression function of Equation (2) cannot be estimated analytically. In this paper, we present a learning approach to directly obtain *f* using deep learning. We represent *f* using a Convolutional Neural Network (CNN) which is learned end-to-end from the microphone signals. In our system, we assume that the positions of microphones are fixed. We thus drop the requirement of knowing their positions from Equation (2) which is implicitly learned by our network with the following regression problem:

(3) $$s=f_{net}\left(x_{1},\ldots ,x_{M}\right),$$

where $f_{net}$ denotes the function that is represented by the CNN with a topology that is described next.

### 3.2. Network Topology

**Figure 1:**
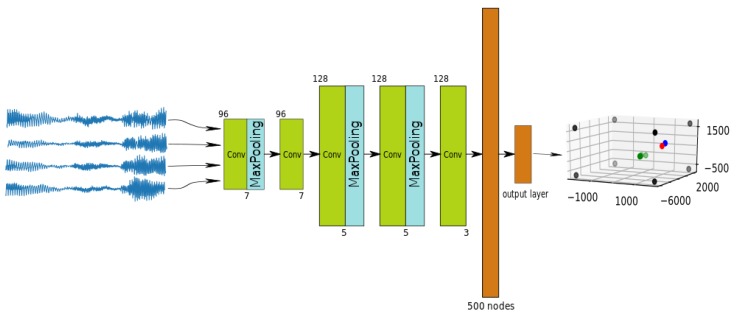
Used network topology.

The topology of our neural network is shown in Figure 1. It is based on two phases: filter-and-sum enhancement by means of 1D convolutional FIR filters, followed by a standard fully connected network. We believe that this architecture is well suited for audio analysis, especially when the window size is fixed, as it is in our case.

The network is composed of five one-dimensional, convolutional blocks and two fully connected blocks. In accordance with Equation (3), the network inputs are the set of windowed signals from the microphones, and the network output is the estimated position of the acoustic source.

Table 1 shows the sizes and number of convolutional filters in the proposed network topology. We used filters of size 7 (layers 1 and 2), size 5 (layers 3 and 4) and size 3 (layer 5). The number of filters is 96 in the first two convolutional layers and 128 in the rest. As seen in Figure 1, some of the layers are equipped with *MaxPooling* filters with the same pool size as their corresponding convolutional filters. The last two layers are fully-connected layers—one hidden with 500 nodes and the output layer. The activation functions of all layers are “ReLUs” with the exception of the output layer. During training, we included a dropout with a probability of 0.5 in the fully-connected layers to prevent overfitting.

With this topology, the network is also very fast to train end-to-end and to run in small GPUs.

### 3.3. Training Strategy

The amount of available real data included in our experimental setup to properly train the CNN model (see Section 4) was, in general, limited. To cope with this problem, we propose a training strategy comprising two steps:

*Step 1*. The network is trained with semi-synthetic data. Close-talk speech recordings and a set of randomly generated source positions are used to generate simulated versions of the signals captured by a set of microphones that share the same geometry as that of the environment used in the real data recordings. Additional considerations on the acoustic behavior of the target environment (specific noise types, noise levels, etc.) are also taken into account to generate the data. A dataset of this type can virtually be made as big as required to train a network.
*Step 2*. The network is fine-tined with real data. The network is trained on a reduced subset of the database captured in the target physical environment using the weights obtained in Step 1 for initialization.

#### 3.3.1. Semi-Synthetic Dataset Generation

In this step, audio signals are extraced from any available close-talk (anechoic) corpus and used to generate semi-synthetic data. There are many available datasets that are suitable for this task (freely or commercially distributed). Our semi-synthetic dataset can thus be made as big as required to train the CNN.

For this task, *Q* position vectors are randomly generated ($q_{i}={\left(q_{i,x}\;\;q_{i,y}\;\;q_{i,z}\right)}^{⊤}$) with i=1,…,Q of the acoustic source using a uniform distribution that covers the physical space (room) that will be used.

The loss function used to train the network is the mean squared error between the estimated position given by the network ($s_{i}$) and the target position vector ($q_{i}$). It follows the expression

(4) $$L\left(\Theta \right)=\frac{1}{Q}\sum_{i=1}^{Q}{\left|q_{i}-s_{i}\right|}^{2},$$

where Θ represents the weights of the network. Equation (4) is minimized as a function of the unknown weights using iterative optimization based on the Stochastic Gradient Descent (SGD) algorithm [63]. The target weights (θ∈Θ) are finally obtained once a termination criterion is met in the optimization. More details about the training algorithm are given in Section 4.

In order to realistically simulate the signals received in the microphones from a given source position, we have to consider two main issues:

- Signal propagation considerations: This is affected by the impulse response of the target room. Different alternatives can be used to simulate this effect, such as convolving the anechoic signals with real room impulse responses, such as in Ref. [60], which can be difficult to acquire for general positions in big environments, or by using room response simulation methods, such as the image method [64] used in Ref. [65] for this purpose.
- The acoustic noise conditions of the room and the recording process conditions: These can result from additional equipment (computers, fans, air conditioning systems, etc.) present in the room, and from problems in the signal acquisition setup. They can be addressed by assuming additive noise conditions and selecting the noise type and acoustic effects that should be preferably estimated in the target room.

In our case, and regarding the first issue, we decided to use the simplest approach as our initial alternative, just taking into account the propagation delay from the source position to each of the microphones which depends on their relative positions and the speed of sound in the room.

Our simulation model does not consider other effects, such as reverberation of the signals in the room or other environmental noise conditions. We thus do not require more specific knowledge about the room, such as the positions and materials of the walls and furniture.

$N_{s_{i}}=f_{s}\frac{d_{i}}{c}$ denotes the number of samples that are required to shift a signal to simulate the arrival delay suffered at microphone *i*, where $f_{s}$ is the sampling frequency of the signal, $d_{i}$ is the Euclidean distance between the acoustic source and the *i*th microphone, and *c* is the speed of sound in air (c=343 m/s in a room at 20 ${}^{\circ }$C). In general, $N_{s_{i}}$ is not an integer number. Thus, a method to simulate sub-sample shifts in the signal is required. In order to implement the delay from $N_{s_{i}}$ on $x_{pc}$ (the windowed signal of *N* samples from the close-talk dataset) to obtain $x_{i}$, the following transformationis used:

(5) $$X_{pc}=F\left\{x_{pc}\right\}\;\;x_{i}=A_{i}\left(F^{-1}\left\{X_{pc}⊙D_{s_{i}}\right\}\right),with\;\;D_{s_{i}}=\left(1,e^{-j\frac{2\pi N_{s_{i}}}{N}},e^{-j\frac{4\pi N_{s_{i}}}{N}},\ldots ,e^{-j\frac{\left(N-1\right)2\pi N_{s_{i}}}{N}}\right)$$

where $x_{pc}$ is first transformed into the frequency domain ($X_{pc}$) using the *Discrete Fourier Transform* operator (F), and ⊙ is the element-wise product. Then, its phase is changed according to $N_{s_{i}}$ by the phase vector $D_{s_{i}}$, and the signal is transformed back into time domain $x_{i}$ using the *Inverse Discrete Fourier Transform* operator $F^{-1}$. $A_{i}$ is an amplitude factor that is applied to the signal that follows an uniform random distribution, and it is different for each microphone ($A_{i}\in \left[0.01,0.03\right]$ in the experimental setup described in Section 4). We used random amplitudes because we explicitly wanted the network to focus on phase or time-delay differences between the microphones. It was intended that these random amplitudes would take away the effects of the directionality of the microphones, and this is so because we assumed that they had omnidirectional responses (as they were in our experimental setup).

Regarding the second issue, we simulated noise and disturbances in the signals arriving to the microphones so that the signal-to-noise ratio and the spectral content of the signals were as similar as possible to those found in the real data. In order to provide an example of the methodology followed, this section refers to the particular case of the IDIAP room (see Section 4.1.1) that was used in our real data experiments and the Albayzin Phonetic Corpus (see Section 4.1.2) that was used for synthetic data generation.

In the IDIAP room, a spectrogram-based analysis showed that the recordings were contaminated with a tone at around 25 Hz in the spectrum which does not appear in anechoic conditions. This was probably the result of room equipment, namely, electrical noise generated in the recording hardware setup. We determined that the frequency of this tone actually varied in a range between 20 Hz and 30 Hz. So, in the synthetic data generation process, we *contaminated* the signals from the phonetic corpus with an additive tone of a random frequency in this established range, and we also added white Gaussian noise in accordance with the expression:

(6) $$x_{pc_{new}}\left[n\right]=x_{pc}\left[n\right]+k_{s}\sin\left(2\pi f_{0}n/f_{s}+\phi_{0}\right)+k_{\eta }\eta_{wgn}\left[n\right],$$

where $k_{s}$ is a scaling factor for the contaminating tone signal (similar to the tone amplitude found in the target room recordings, 0.1 in our case), $f_{0}\in \left[20,30\right]$ Hz, $\phi_{0}\in \left[0,\pi \right]rad$, $\eta_{wgn}$ is a white Gaussian noise signal, and $k_{\eta }$ is a noise scaling factor to generate signals with a similar Signal to Noise Ratio (SNR) to that found in the target room recordings.

After this procedure was applied, the semi-synthetic signal data set was be ready to be used in the neural network training procedure.

#### 3.3.2. Fine Tuning Procedure

The previous step took care of reproducing the simple acoustic characteristics of the microphone array configuration and the presence of specific types and levels of additive noises, but there are other phenomena, like the presence of diffuse noise and multi-path propagation and reverberation, which are more complex to simulate. In order to introduce these acoustic characteristics from the target physical environment, we carried out a fine tuning procedure on the network model, using a short amount of real recorded data in the target room.

The fine tuning procedure consisted of training the network with some of the sequences recorded in the real environment after initializing the network weights obtained from the training phase with the semi-synthetic data.

Although there are other methods, such as the one proposed in Ref. [5], where an unsupervised DNN has been implemented for the adaptation of parameters to unknown data, we believe that our fine tuning process is adequate, because, firstly, it is a supervised process with which a better performance is expected to be obtained and, secondly, because not all the sequences of the test data set are used—only a few are used for the fine tuning process, saving the rest for the test phase.

## 4. Experimental Work

In this section, we describe the datasets used in both steps of the training strategy described in Section 3.3, and the details associated with them. We then define the general conditions of the experimental setup and the error metrics used for comparing our proposal with other state-of-the-art methods, and finally, we present our experimental results, starting from the baseline performance that we aimed to improve.

### 4.1. Datasets

#### 4.1.1. IDIAP AV16.3 Corpus: For Testing and Fine Tuning

We evaluated our proposal with the audio recordings from the AV16.3 database [66], an audio-visual corpus recorded in the *Smart Meeting Room* of the IDIAP research institute in Switzerland. We also used the physical layout of this room for our semi-synthetic data generation process.

**Figure 2:**
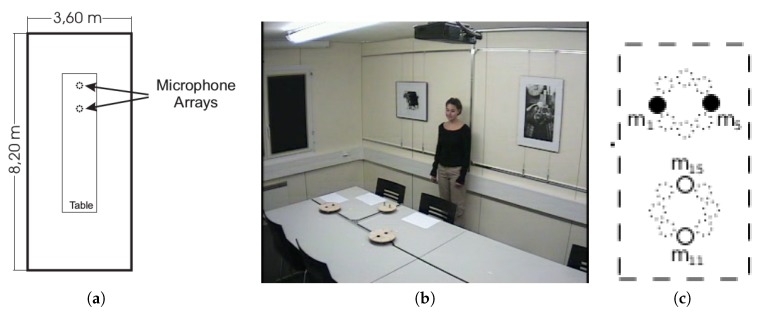
(**a**) Simplified top view of the *IDIAP Smart Meeting Room*; (**b**) a real picture of the room extracted from a video frame; (**c**) microphone setup used in this proposal.

The *IDIAP Smart Meeting Room* is a 3.6 m × 8.2 m × 2.4 m rectangular room with a centrally located rectangular table that measures 4.8 m × 1.2 m. On the table’s surface, there are two circular microphone arrays of 0.1 m radius, each of them composed by eight regularly distributed microphones, as shown in Figure 2. The centers of both arrays are separated by a distance of 0.8 m. The middle point between them is considered to be the origin of the coordinate reference system. A detailed description of the meeting room can be found in Ref.  [67].

The dataset is composed of several sequences of recordings, synchronously sampled at 16 KHz, with a wide range of experimental conditions in the number of speakers involved and their activity. Some of the available audio sequences are assigned a corresponding annotation file that contains the real ground truth positions (3D coordinates) of the speaker’s mouth at every time frame in which that speaker was talking. The segmentation of acoustic frames with speech activity was first checked manually at certain time instances by a human operator in order to ensure its correctness, and later, this was extended to cover the rest of the recording time by means of interpolation techniques. The frame shift resolution was defined to be 40 ms. The complete dataset is fully accessible online at Ref. [68].

In this paper, we focused on the annotated sequences of this dataset that featured a single speaker, whose main characteristics are shown in Table 2. This allowed us to directly compare our performance with the method presented in Ref. [35] that was proven to achieve better results than the *SRP-PHAT* algorithm. Note that the first three sequences involved a speaker who remained static while speaking at different positions, and the last two ones involved a moving speaker; all of the speakers were different. We refer to these sequences as s01, s02, s03, s11, and s15 for brevity.

Depending on the sequence being considered, the distances between the speakers and the microphone arrays had maximum, minimum, and average values in the intervals (209, 243) cm, (46, 156) cm and (118, 192) cm, respectively.

#### 4.1.2. Albayzin Phonetic Corpus: For Semi-Synthetic Dataset Generation

The Albayzin Phonetic Corpus [69] consists of three sub-corpora of 16 kHz 16 bits signals, recorded by 304 Castilian Spanish speakers in a professional recording studio using high quality, close-talk microphones.

We used this dataset to generate semi-synthetic data, as described in Section 3.3.1. From the three sub-corpora, we only used the so-called *phonetic corpus* [70], which is composed of 6800 utterances of phonetically balanced sentences. This phonetical balance characteristic makes this dataset perfect for generating our semi-synthetic data, as it covers all possible acoustic contexts.

### 4.2. Training and Fine Tuning Details

In the semi-synthetic dataset generation procedure, described in Section 3.3.1, we generated random positions ($q_{i}$) with uniformly distributed values in the following intervals: $q_{i,x}\in \left[0,3.6\right]$ m, $q_{i,y}\in \left[0,8.2\right]$ m and $q_{i,z}\in \left[0.92,1.53\right]$ m, which corresponded to the possible distributions of the speaker’s mouth positions in the *IDIAP* room [66].

Regarding the optimization strategy for the loss function described by Equation (4), we employed the *ADAM* [71] optimizer (variant of SGD with a variable learning rate) along 200 epochs with a batch size of 100 samples. The learning rate of the *ADAM* optimizer was fixed at $\alpha =10^{-3}$, and the other parameters were set with the recommended values in accordance with Ref. [71] ($\beta_{1}=0.9$, $\beta_{2}=0.99$ and $\epsilon =10^{-8}$). A total of 7200 different frames of input data per epoch were randomly generated during the training phase, and another 800 were generated for validation.

The experiments were performed with three different window lengths (80 ms, 160 ms, and 320 ms), and the training phase was run once per window length, obtaining three different network models. In each training session, 200 audio recordings were randomly chosen and 40 different windows were randomly extracted from each. In the same way, 200 acoustic source position vectors ($q_{i}$) were randomly generated, so each position generated 40 windows of the same signal.

For the fine tuning procedure described in Section 3.3.2, we mainly used sequences s11 and s15 that feature a speaker moving in the room while speaking, as well as sequences s01, s02 and s03 in a final experiment. The *ADAM* optimizer was also used for fine tuning. In this case, we fixed the learning rate at $\alpha =10^{-4}$, while the rest of the parameters were set to the recommended values.

As described in Section 4.6, we also carried out experiments to assess the relevance of adding additional sequences (s01, s02, and s03) to complement the fine tuning data provided by s11 and s15. We also refer to gender and height issues in the fine tuning and evaluation data.

### 4.3. Experimental Setup

In our experiments, sequences s01, s02, and s03 were used to test the performance of our network, and as explained above, to complement sequences s11 and s15 for fine tuning.

In this work, we used a simple microphone array configuration to evaluate our proposal in a resource-restricted environment, as was done in Ref. [35]. In order to do so, we used four microphones (numbers 1, 5, 11, and 15, out of the 16 available in the AV16.3 dataset), grouped in two microphone pairs. This configuration of four microphones is the same as that selected in Ref. [35] to provide two orthogonal microphone pairs. The selected microphone pair configurations are shown in Figure 2c, in which microphones of the same color are considered to belong to the same microphone pair. We assessed the results related to the length of the acoustic frame for 80 ms, 160 ms, and 320 ms to precisely assess to what extent the improvements were consistent with varying acoustic time resolutions.

The main interest of our experimental work was to assess whether the end-to-end CNN based approach (that we will refer to as CNN) could be competitive with other traditional localization methods. We compared this CNN approach with the standard *SRP-PHAT* method and the recent strategy proposed in Ref. [35] that we refer to as GMBF. This GMBF method is based on fitting a generative model to the GCC-PHAT signals using sparse constraints, and it was associated with significant improvements over *SRP-PHAT* in the *IDIAP* dataset [35,72]. The GMBF fitting procedure does not require training, as opposed to the CNN approach. We also compare our method with another very recently published ASL strategy based on a Convolutional Recurrent Neural Network (CRNN) [18], with a similar scope.

After providing the baseline results of the comparison between *SRP-PHAT*, GMBF, and our proposal without applying the fine tuning procedure, we then describe three experiments, that we briefly summarize here:

- In the first experiment, we evaluate the performance improvements when using a single sequence for the fine tuning procedure.
- In the second experiment, we evaluate the impact of adding an additional fine tuning sequence.
- In the third experiment, we evaluate the final performance improvements when also adding static sequences to the refinement process.

After these experiments, we evaluate the differences between the semi-synthetic training plus the fine tuning approach versus just training the network from scratch, to validate the contribution of the fine tuning strategy.

Finally, we provide a comparison between our proposal and that described in Ref. [18], for which the authors kindly provided the source code [73].

### 4.4. Evaluation Metrics

Our CNN based approach yielded a set of spatial coordinates $s_{k}={\left(s_{k,x}\;\;s_{k,y}\;\;s_{k,z}\right)}^{⊤}$ that are estimations of the current speaker’s position as time instant *k*. These position estimates were compared, by means of the Euclidean distance, to the ones labeled in a transcription file containing the real positions, $s_{k_{GT}}$ (*ground truth*), of the speaker.

We evaluated performance by adopting the same metric used in Ref. [35] and developed under the CHIL project [74]. It is known as MOTP (*Multiple Object Tracking Precision*) and is defined as

(7) $$MOTP=\frac{\sum_{k=1}^{N_{P}}{\left|s_{k_{GT}}-s_{k}\right|}^{2}}{N_{P}},$$

where $N_{P}$ denotes the total number of position estimations along time, $s_{k}$ the estimated position vector, and $s_{k_{GT}}$ is the labeled ground truth position vector.

We compared our experimental results, and that of the GMBF method, with that of *SRP-PHAT* by measuring the relative improvement in MOTP which is defined as follows:

(8) $$\Delta_{r}^{MOTP}=100\frac{MOTP_{SRP-PHAT}-MOTP_{proposal}}{MOTP_{SRP-PHAT}}\left[\%\right].$$

### 4.5. Baseline Results

The baseline results for sequences s01, s02 and s03 are shown in Table 3 as well as the evaluated time window sizes (in all the tables showing results in this paper, **bold font** highlights the best ones for a given data sequence and window length). The table shows the results achieved by the *SRP-PHAT* standard algorithm strategy (column SRP), the alternative described in Ref. [35] (column GMBF), and the proposal in this paper without applying the fine tuning procedure (column CNN). We also show the relative improvements of GMBF and CNN compared with SRP-PHAT.

The main conclusions from the baseline results are as follows:

- The MOTP values improved as the frame size increased, as expected, given that better correlation values will be estimated for longer window signal lengths. The best MOTP values for the standard SRP-PHAT algorithm were around 69 cm, and for the GMBF, around 48 cm.
- The average MOTP value for the standard SRP-PHAT algorithm was between 76 cm and 96 cm, and for the GMBF, it was between 59 cm and 78 cm.
- The GMBF strategy, as described in Ref. [35], achieves very relevant improvements compared with SRP-PHAT, with average relative improvements of around 20% and peak improvement values of almost 30%.
- Our CNN strategy, which, at this point, is only trained with semi-synthetic data, was shown to be very far from reaching SRP-PHAT or GMBF in terms of performance. This result leads us to think that there are other effects that are only present in real data such as reverberation that are affecting the network, as they have not been properly modeled in the training data. This could be addressed by introducing simulation algorithms that can model room propagation effects (such as the image source method [64,75]) to generate more realistic semi-synthetic data. This will be evaluated in future work.

So, given the discussion above, we decided to apply the fine tuning strategy discussed in Section 3.3.2 with the experimental details described in Section 4.2. The results shown in Table 3 were compared with those obtained by our CNN method under different fine tuning (and training) conditions, and they are described below.

### 4.6. Results and Discussion

The first experiment in which we applied the fine tuning procedure used s15 as the fine tuning subset.

Table 4 shows the results obtained by GMBF (column GMBF) and CNN with this fine tuning strategy (column CNNf15 ). The results in the table shown that CNNf15 is, most of the time, better than the *SRP-PHAT* baseline (except in two cases for s03 in which there was a slight degradation). The average performance showed a consistent improvement in CNNf15 compared with SRP-PHAT, between 1.8% and 11.3%. However, CNNf15 was still behind GMBF in all cases but one (for s02 and 80 ms).

Our conclusion is that the fine tuning procedure is able to effectively complement the trained models from synthetic data, leading to results that outperform SRP-PHAT. This is specially relevant due to the following points.

- The amount of fine tuning data is limited (only 36 s, corresponding to 436 frames, as shown in Table 2), thus opening the path to further improvements with a limited data recording effort.
- The speaker used for fine tuning was mostly moving while speaking, while in the testing sequences, the speakers are static while speaking. This means that the fine tuning material included far more active positions than the testing sequences, and the network was able to extract the relevant information for the tested positions.
- The improvements obtained by our CNN decrease for longer signal window sizes suggest that the speake’sr speed (and thus, the displacement of the speaker across the signal window) might be having an impact on the results. We evaluated the average speed of the speakers for the moving speaker sequences, 0.72 m/s for s11, and 0.48 m/s for seq15. They do not seem to have a significant relevant impact on position estimation. We also evaluated the source displacement distribution within individual signal frames across the different sequences. The average displacement distances were 4–6 cm for the 80 ms window, 7–11 cm for the 160 ms window and 15–20 cm for the 320 ms window. When we considered the maximum displacement distances, these values turned out to be 7–27 cm for the 80 ms window, 14–34 cm for the 160 ms window, and 28–46 cm for the 320 ms case. These displacements could have a visible impact on the results, and they might be the reason for the lower improvements achieved by our method for longer window sizes.
- The speaker used for fine tuning was male, and the obtained results for male speakers (sequences s01 and s03) and the female one (sequence s02) do not seem to show any gender-dependent bias, which means that the gender issue does not seem to play a role in the adequate adaptation of the network models.

In spite of the relevant improvements with the fine tuning approach, they are still far from making this method suitable for further competitive exploitation in the ASL scenario (provided we have the GMBF alternative), so we next aimed to increase the amount of fine tuning material.

In our third experiment, we applied the fine tuning procedure using an additional *moving speaker* sequence, that is, by including s15 and s11 in the fine tuning subset.

Table 5 shows the results obtained by GMBF and CNN after fine tuning with s15 and s11 (CNNf15+11 columns). In this case, additional improvements over using only s15 for fine tuning occurred, and there was only one case in which CNNf15+11 did not outperform SRP-PHAT (with a marginal degradation of −0.3%).

The CNN based approach again showed an average, consistent improvement compared with SRP-PHAT of between 7.5% and 16.2%.

In this case, the newly added sequence (s11, with a duration of only 33 s) for fine tuning corresponded to a randomly moving male speaker, and the results show that its addition contributed to further improvements in the CNN based proposal, but it was still behind GMBF in all cases but two, but with results getting closer. This suggests that a further increment in the fine tuning material should be considered.

Our last experiment consisted of fine tuning the network, including additional static speaker sequences. To assure that the training (including fine tuning) and testing material were fully independent, we fine-tuned with s15, s11 and with the static sequences that were not tested in each experiment run, as shown in Table 6.

Table 7 shows the results obtained for this fine tuning scenario. The main conclusions were as follows:

- The CNN based method exhibited much better average behavior than GMBF for all window sizes. The average absolute improvement against SRP-PHAT for the CNN was more than 10 points higher than for GMBF, reaching 32.8% in the CNN case and 22.9% in GMBF.
- Considering the individual sequences, CNN was shown to be significantly better than GMBF for sequences s01 and s02, and slightly worse for s03.
- Considering the best individual result, the maximum improvement for the CNN was 41.6% (s01, 320 ms), while the top result for GMBF was 29.9% (s03, 320 ms).
- The effect of adding static sequences was shown to be beneficial, as expected, provided that the acoustic tuning examples were generated from similar, but not identical, positions, as the speakers had varying heights and their positions in the room were not strictly equal from sequence to sequence.
- The improvements obtained were significant and came at the cost of additional fine tuning sequences. However, this extra cost was still reasonable, as the extra fine tuning material is of limited duration, around 400 s on average (6.65 min).

**Figure 3:**
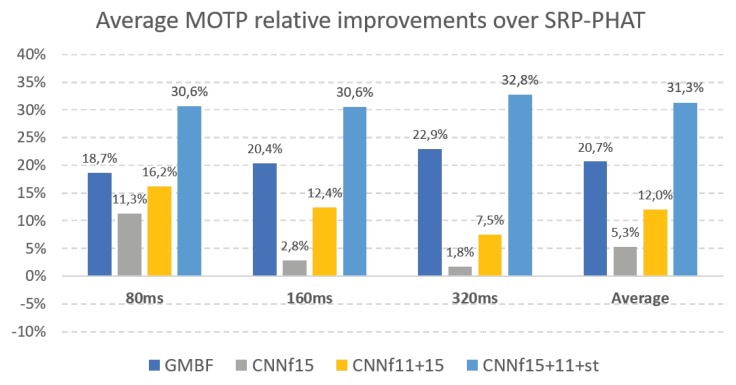
MOTP relative improvements over SRP-PHAT for GMBF and CNN using different fine tuning subsets (for all window sizes).

Finally, to summarize, Figure 3 shows the average MOTP relative improvements over *SRP-PHAT* obtained by our CNN proposal using different fine tuning subsets and its comparison with the GMBF results for all of the signal window sizes.

From the results obtained by our proposal, it is clear that the highest contribution to the improvements from the bare CNN training was the fine tuning procedure with limited data (CNNf15, compare Table 3 and Table 4), while the use of additional fine tuning material consistently improved the results (Table 5 and Table 7). It is again worth noticing that these improvements were consistently independent of the height and gender of the considered speaker and whether there was a match or not between the static or dynamic activity of the speakers being used in the fine tuning subsets. This suggests that the network actually learns the acoustic cues that are related to the localization problem. Thus, we conclude that our proposal is a suitable and promising strategy for solving the ASL task.

### 4.7. Validation of the Fine Tuning Strategy

When comparing the results of Table 3 and Table 4, and given the large improvement when applying the fine tuning strategy, it could be assumed that the initial training with semi-synthetic data is limited. Based on this argument, we ran additional training experiments in which we just trained the network *from scratch* with the same sequences used in the experiments shown in Table 4, Table 5 and Table 7, with the objective of assessing the actual effect of combining semi-synthetic training and fine tuning versus just training with real room data. The training strategy and parameters were the same as those used when training the network from semi-synthetic data, and they are described in Section 4.2.

Table 8 shows a comparison between these two options using different sequences. The figures shown are the average values across all testing sequences for each case. The results for the training from scratch approach are included in column tr−sc, and those for our proposed combined semi-synthetic training and fine tuning strategy are included in column tr+ft.

When using s15 in the training and fine tuning procedures (first row of Table 8), the average improvement of the tr+ft approach varied between 1.8% and 11.3% with an average improvement over all window lengths of 5.3%, while the tr−sc average improvement varied between −20.6% and 4.3% with an average value of −7.0%.

When using s15 and s11 in the training and fine tuning procedures (second row of Table 8), the average improvement of the tr+ft approach varied between 7.5% and 16.3% with an average improvement over all window lengths of 12.0%, while the tr−sc average improvement varied between −29.4% and 0.6% with an average value of −12.1%.

Finally, when using the sequences described in Table 6 (third row of Table 8), the average improvement of the tr+ft approach varied between 30.6% and 32.8% with an average improvement over all window lengths of 31.3%, while the tr−sc average improvement varied between 2.3% and 17.3% with an average value of 11.0%.

So, in all of the evaluated cases, the combined semi-synthetic training and fine tuning approach clearly outperformed the training from scratch strategy, thus validating our methodology.

### 4.8. Comparison with Deep Learning Methods

In this section, we also provide a comparison between our proposal and a recent deep learning ASL method known as SELDnet [18], for which the source code is available in Ref. [73]. SELDnet is a CRNN architecture that uses the signal spectrum of the audio signals as inputs (phase and magnitude components of the spectrogram calculated on each audio channel) and is able to deal with multiple overlapping sound events.

SELDNet generates two different outputs:

- Classification output: The first output of the SELDnet is able to classify the sound events among a list of classes for each consecutive frame in the input audio signals.
- Regression output: The second output estimates the DOA vector detected on each of the consecutive frames in the audio input. This vector is parametrized as the *x*, *y*, and *z* axis coordinates of the DOA on a unit sphere around the microphone, which is claimed to lead to a network that learns better than one that uses a parametrization based on angles.

As suggested by the authors, we used the default values of the SELDnet design parameters regarding the feature extraction, network model, and training process, and in order to carry out the comparison with our method, the following issues were taken into account:

- SELDnet uses an audio window of size $w_{s}$ for each microphone and extracts consecutive overlapped frames to compute the spectral components that are used as inputs. To compare this with our network, we performed experiments with different values of $w_{S}$: 80 ms, 160 ms, and 320 ms.
- Due to the fact that we used sequences of audio where only a single speaker appeared simultaneously, we assigned the same label (“speech”) to all the audio windows used for training.
- We needed SELDnet to infer the x,y,z coordinates of the target source, instead of the DOA vector. This only required us to change the target output during training, as the network model does not change it at all. Our spatial coordinates were also normalized in the interval [−1,1] which is compatible with the regression output of the SELDnet. The final output coordinates were again denormalized back to metric coordinates to proceed with the MOTP calculations.
- We followed the same experimental procedure as in our proposal (initial semi-synthetic training followed by fine tuning) in a resource-restricted scenario using only two microphone pairs. The experimental conditions were those for which we got the best performance (included in Table 7), that is, using the testing and fine tuning sequences described in Table 6.

Table 9 shows the relative improvements of the proposal in Ref. [18] (column SELDnet) and our CNN approach (column CNNf15+11+st) over SRP-PHAT.

It can be clearly seen that the SELDnet produced worse results than our CNN approach in terms of localization accuracy and it actually performed worse than the standard SRP-PHAT algorithm.

## 5. Conclusions

In this paper, we presented an audio localization CNN that is trained end-to-end from the raw audio signals to the source position. We showed that this method is very promising, as it outperforms the state-of-the-art methods [35,72] and those using *SRP-PHAT* when sufficient fine tuning data is available. It also performed better than a very recent proposal based on CRNNs. In addition, our experiments show that the CNN method exhibits good resistance against varying gender of the speaker and different window sizes compared with the baseline methods.. Given that the amount of data recordings for audio localization is limited at the moment, we proposed in the paper to first train the network using semi-synthetic data followed by fine tuning using a small amount of real data. This has been a common strategy in other fields to prevent overfitting, and we showed ithat it significantly improves the system performance as compared with training the network from scratch using real data.

In a future line of work, we plan to improve the generation of semi-synthetic data including reverberation effects and testing, in detail, the effects of gender and language on the system’s performance. In addition we plan to include more real data by developing a large corpus for audio localization that will be made available to the scientific community for research purposes. Also, we will address the multiple source scenario, and an extensive evaluation will be carried out to asses the impact of the proposal on more complex and varying acquisition scenarios (comprising a higher number of microphone pairs and different rooms). This extensive evaluation will include explicit comparisons with other DNN-based proposals in the ASL task.

## Figures and Tables

**Table 1:** Network convolutional layers summary.

Block	Filters	Kernel
Convolutional block 1	96	7
Convolutional block 2	96	7
Convolutional block 3	128	5
Convolutional block 4	128	5
Convolutional block 5	128	3

**Table 2:** *IDIAP Smart Meeting Room* used sequences.

Sequence (abbr.)	Average Speaker Height (cm) *	Duration (s)	Number of Ground Truth Frames	Description
seq01-1p-0000 (s01)	54.3	208	2248	A single male speaker, static while speaking, at each of the 16 locations. The speaker is facing the microphone arrays.
seq02-1p-0000 (s02)	62.5	171	2411	A single female speaker, static while speaking, at each of 16 locations. The speaker is facing the microphone arrays.
seq03-1p-0000 (s03)	70.3	220	2636	A single male speaker, static while speaking, at each of the 16 locations. The speaker is facing the microphone arrays.
seq11-1p-0100 (s11)	53.5	33	481	A single male speaker, making random movements while speaking, and facing the arrays.
seq15-1p-0100 (s15)	79.5	36	436	A single male speaker, walking around while alternating speech and long silences. No constraints

* The average speaker height is referenced to the system’s coordinates and refers to the speaker’s mouth height.

**Table 3:** Baseline results for the SRP-PHAT strategy (column SRP), the one in [35] (column GMBF), and the Convolutional Neural Network (CNN) trained with synthetic data without applying the fine tuning procedure (column CNN) for sequences s01, s02 and s03 for different window sizes. Relative improvements compared to SRP-PHAT are shown below the MOTP (Multiple Object Tracking Precision) values.

	80 ms			160 ms			320 ms		
	SRP	GMBF	CNN	SRP	GMBF	CNN	SRP	GMBF	CNN
s01 $\begin{array}{c} MOTP(m)\\ \Delta_{r}^{MOTP} \end{array}$	1.020	0.795	1.615	0.910	0.686	1.526	0.830	0.588	1.464
		22.1%	−58.3%		24.6%	−67.7%		29.1%	−76.4%
s02 $\begin{array}{c} MOTP(m)\\ \Delta_{r}^{MOTP} \end{array}$	0.960	0.864	2.124	0.840	0.759	1.508	0.770	0.694	1.318
		10.0%	−121.3%		9.6%	−79.5%		9.9%	−71.2%
s03 $\begin{array}{c} MOTP(m)\\ \Delta_{r}^{MOTP} \end{array}$	0.900	0.686	1.559	0.770	0.563	1.419	0.690	0.484	1.379
		23.8%	−73.2%		26.9%	−84.3%		29.9%	−99.9%
Average $\begin{array}{c} MOTP(m)\\ \Delta_{r}^{MOTP} \end{array}$	0.957	0.778	1.763	0.836	0.666	1.481	0.760	0.585	1.385
		18.7%	−84.3%		20.4%	−77.1%		22.9%	−82.3%

**Table 4:** Results for the stratgy in [35] (column GMBF); and the CNN that was fine-tuned with sequence s15 (column CNNf15).

	80 ms		160 ms		320 ms	
	GMBF	CNNf15	GMBF	CNNf15	GMBF	CNNf15
s01 $\begin{array}{c} MOTP(m)\\ \Delta_{r}^{MOTP} \end{array}$	0.795	0.875	0.686	0.833	0.588	0.777
	22.1%	14.2%	24.6%	8.5%	29.1%	6.4%
s02 $\begin{array}{c} MOTP(m)\\ \Delta_{r}^{MOTP} \end{array}$	0.864	0.839	0.759	0.801	0.694	0.731
	10.0%	12.6%	9.6%	4.6%	9.9%	5.1%
s03 $\begin{array}{c} MOTP(m)\\ \Delta_{r}^{MOTP} \end{array}$	0.686	0.835	0.563	0.806	0.484	0.734
	23.8%	7.2%	26.9%	-4.7%	29.9%	-6.4%
Average $\begin{array}{c} MOTP(m)\\ \Delta_{r}^{MOTP} \end{array}$	0.778	0.849	0.666	0.813	0.585	0.746
	18.7%	11.3%	20.4%	2.8%	22.9%	1.8%

**Table 5:** Relative improvements over SRP-PHAT for the strategy presented in Ref. [35] (columns GMBF) and the CNN fine-tuned with sequences s15 and s11 (columns CNNf15+11).

	80 ms		160 ms		320 ms	
	GMBF	CNNf15+11	GMBF	CNNf15+11	GMBF	CNNf15+11
s01 $\begin{array}{c} MOTP(m)\\ \Delta_{r}^{MOTP} \end{array}$	0.795	0.805	0.686	0.750	0.588	0.706
	22.1%	21.1%	24.6%	17.6%	29.1%	14.9%
s02 $\begin{array}{c} MOTP(m)\\ \Delta_{r}^{MOTP} \end{array}$	0.864	0.809	0.759	0.716	0.694	0.712
	10.0%	15.7%	9.6%	14.8%	9.9%	7.5%
s03 $\begin{array}{c} MOTP(m)\\ \Delta_{r}^{MOTP} \end{array}$	0.686	0.792	0.563	0.732	0.484	0.692
	23.8%	12.0%	26.9%	4.9%	29.9%	−0.3%
Average $\begin{array}{c} MOTP(m)\\ \Delta_{r}^{MOTP} \end{array}$	0.778	0.802	0.666	0.732	0.585	0.703
	18.7%	16.2%	20.4%	12.4%	22.9%	7.5%

**Table 6:** Fine tuning material used in the experiment corresponding to columns CNNf15+11+st in Table 7.

Test Sequence	Fine Tuning Sequences
seq01	s15 + s11 + s02 + s03
seq02	s15 + s11 + s01 + s03
seq03	s15 + s11 + s01 + s02

**Table 7:** Relative improvements over SRP-PHAT for the strategy presented in Ref. [35] (column GMBF) and the CNN fine-tuned with the sequences described in Table 6 (column CNNf15+11+st).

	80 ms		160 ms		320 ms	
	GMBF	CNNf15+11+st	GMBF	CNNf15+11+st	GMBF	CNNf15+11+st
s01 $\begin{array}{c} MOTP(m)\\ \Delta_{r}^{MOTP} \end{array}$	0.795	0.607	0.686	0.540	0.588	0.485
	22.1%	40.5%	24.6%	40.7%	29.1%	41.6%
s02 $\begin{array}{c} MOTP(m)\\ \Delta_{r}^{MOTP} \end{array}$	0.864	0.669	0.759	0.579	0.694	0.545
	10.0%	30.3%	9.6%	31.1%	9.9%	29.2%
s03 $\begin{array}{c} MOTP(m)\\ \Delta_{r}^{MOTP} \end{array}$	0.686	0.707	0.563	0.617	0.484	0.501
	23.8%	21.4%	26.9%	19.9%	29.9%	27.4%
Average $\begin{array}{c} MOTP(m)\\ \Delta_{r}^{MOTP} \end{array}$	0.778	0.664	0.666	0.581	0.585	0.511
	18.7%	30.6%	20.4%	30.6%	22.9%	32.8%

**Table 8:** Results for the CNN proposal, either trained from scratch (column tr−sc) or using semi-synthetic training + fine tuning (column tr+ft), for different training/fine tuning sequences.

tr−sc/tr+ft Sequences		80 ms		160 ms		320 ms	
		tr−sc	tr+ft	tr−sc	tr+ft	tr−sc	tr+ft
s15	$\begin{array}{c} MOTP(m)\\ \Delta_{r}^{MOTP} \end{array}$	0.915	0.849	0.875	0.813	0.916	0.746
		4.3%	11.3%	−4.6%	2.8%	−20.6%	1.8%
s15+s11	$\begin{array}{c} MOTP(m)\\ \Delta_{r}^{MOTP} \end{array}$	0.951	0.802	0.900	0.732	0.983	0.703
		0.6%	16.2%	−7.6%	12.4%	−29.4%	7.5%
Sequences of Table 6	$\begin{array}{c} MOTP(m)\\ \Delta_{r}^{MOTP} \end{array}$	0.791	0.664	0.724	0.581	0.742	0.511
		17.3%	30.6%	13.4%	30.6%	2.3%	32.8%

**Table 9:** Relative improvements over SRP-PHAT for the strategy in [18] (column SELDnet); and the CNN fine-tuned with the sequences described in Table 6 (column CNNf15+11+st).

	80 ms		160 ms		320 ms	
	SELDnet	CNNf15+11+st	SELDnet	CNNf15+11+st	SELDnet	CNNf15+11+st
s01 $\begin{array}{c} MOTP(m)\\ \Delta_{r}^{MOTP} \end{array}$	1.037	0.607	1.039	0.540	1.076	0.485
	−1.7%	40.5%	−14.2%	40.7%	−29.6%	41.6%
s02 $\begin{array}{c} MOTP(m)\\ \Delta_{r}^{MOTP} \end{array}$	1.035	0.669	1.003	0.579	0.981	0.545
	−7.8%	30.3%	−19.4%	31.1%	−27.4%	29.2%
s03 $\begin{array}{c} MOTP(m)\\ \Delta_{r}^{MOTP} \end{array}$	1.017	0.707	0.991	0.617	1.020	0.501
	−13.0%	21.4%	−28.7%	19.9%	−47.8%	27.4%
Average $\begin{array}{c} MOTP(m)\\ \Delta_{r}^{MOTP} \end{array}$	1.029	0.664	1.010	0.581	0.585	0.511
	−7.6%	30.6%	−20.7%	30.6%	−34.9%	32.8%
